# In Situ Fluorescent Visualization of the Interfacial Layer of Induced Crystallization in Polyvinyl Chloride

**DOI:** 10.3390/polym16223147

**Published:** 2024-11-12

**Authors:** Zhihang An, Renping Liu, Zhenhao Dai, Jiaping Liu, Jiaying Du, Zhongyi Sheng, Heyang Liu

**Affiliations:** 1 College of Biological & Chemical Engineering, Zhejiang University of Science and Technology, Hangzhou 310023, China; zhihangan@zust.edu.cn (Z.A.); daizhenhao1021@163.com (Z.D.); 17816716137@163.com (J.L.); jyducxy@163.com (J.D.); 2 Zhejiang Wazam New Materials Co., Ltd., Hangzhou 311121, China; 15776548418@163.com; 3 College of Environmental and Natural Resources, Zhejiang University of Science and Technology, Hangzhou 310023, China

**Keywords:** polyvinyl chloride, nucleating agent, induced crystallization, interfacial layer, fluorescent sensors

## Abstract

Despite the remarkable progress in the modification and application of polyvinyl chloride (PVC), developing processing aids for the induced crystallization of PVC and characterizing its interfacial layer remain challenges. Herein, we propose a new polymeric nucleating agent, polyamidea12-graft-styrene–maleic anhydride copolymer (PA12-g-SMA), which possesses high compatibility and crystallinity, effectively improving the crystallinity to 15.1%, the impact strength to 61.03 kJ/m^2^, and the degradation temperature of PVC to 267 °C through a single and straightforward processing step. Additionally, after the introduction of two different fluorescent sensors in PA12-g-SMA and PVC, the interfacial layer of the induced crystallization can be monitored in situ via a confocal laser scanning microscope (CLSM). This study highlights a rare strategy for significantly enhancing the physical properties of rigid PVC through simply adding a polymeric nucleating agent during processing, while also emphasizing the importance of visualizing the interfacial layer to understand various polymer crystallization processes.

## 1. Introduction

Polyvinyl chloride (PVC) ranks among the most extensively utilized plastics globally, owing to its excellent properties such as superior flame retardancy, abrasion resistance, and corrosion resistance [1,2,3]. But PVC is classified as an amorphous polymer because the chlorine atoms in its molecular chain disrupt the symmetry and regularity of the polymer structure [4,5]. The low crystallinity of PVC results in decreased thermal stability, making the material prone to thermal degradation during processing, which subsequently diminishes its mechanical properties [6,7,8]. However, the thermal stabilizers designed to enhance the thermal stability of PVC are often complex organometallic compounds that must be used in substantial quantities, which can adversely affect the material’s mechanical properties [9,10,11,12]. Therefore, improving crystallinity to comprehensively enhance the performance of PVC is both significant and challenging.

The crystallization-inducing effect on PVC by nucleating agents is a key method for enhancing its crystallinity, which subsequently improves both its mechanical properties and thermal stability [13,14,15,16]. Compared to inorganic and traditional organic nucleating agents, polymeric nucleating agents exhibit superior dispersion and compatibility within the PVC matrix, leading to a more effective increase in PVC crystallinity [17,18,19,20]. For instance, the graft copolymer of styrene–maleic anhydride copolymer (SMA) and polyamide6 (PA6) enhances the crystallinity of PVC through isothermal crystallization at 110 °C. However, with a melting point of 197 °C, its processing temperature significantly exceeds the typical thermal degradation temperature of PVC (180 °C), which can lead to severe thermal degradation of the material [21]. Currently, there are almost no reported studies on polymeric nucleating agents that induce crystallization during PVC processing. Consequently, we propose a novel polymeric nucleating agent based on high-crystallinity polyamide12 (PA12), as its melting point of approximately 175 °C is below the typical processing temperature of PVC. This innovative polymeric nucleating agent, which significantly enhances PVC performance in a single processing step, not only pushes the boundaries of conventional PVC nucleating agents but also holds substantial practical significance.

The crystallization-inducing effect of polymeric nucleating agents essentially involves transitioning the PVC molecular chains in their vicinity from a disordered to an ordered arrangement, thereby effectively reducing the nucleation energy [22,23,24,25]. Characterizing the interface layer formed by the diffusion of polymer chains from both components can enhance the direct study of the internal structure of PVC crystals [26,27,28,29]. However, current characterization methods, such as atomic force microscopy (AFM) and polarized optical microscopy (POM), are still unable to achieve this due to interference from micron-sized crystalline processing aids in PVC [30,31,32,33]. Fluorescent sensors are effective tools for observing the crystalline regions of polymers [34,35,36]. Zhou et al. observed the interface between the crystalline and amorphous regions of polylactide after doping with a fluorescent sensor using fluorescence microscopy (FM) [37]. Overall, developing a fluorescent sensor capable of observing the more complex interfacial layer between PVC and polymeric nucleating agents remains a significant challenge, crucial for advancing high-performance PVC.

In this work, a strategy for effectively enhancing the performance of PVC through induced crystallization is proposed, which is achieved by simply adding a novel polymeric nucleating agent through a single and straightforward processing step. Additionally, after modification of the polymeric nucleating agent with fluorescent groups, we develop a rare visualization method for characterizing the interfacial layer between the polymer and induced crystallization.

**Figure 1:**
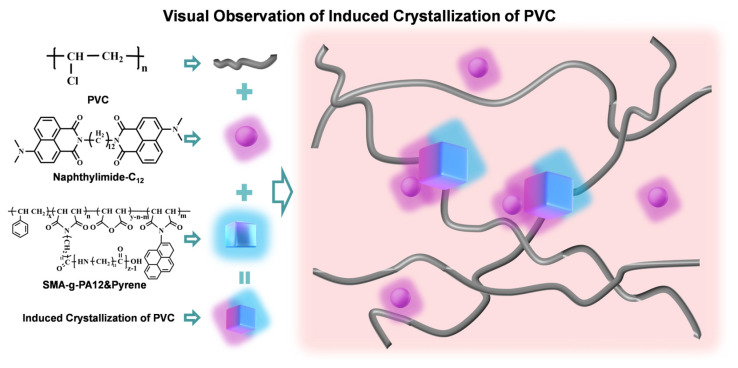
Visual observation of the interfacial layer of induced crystallization was achieved through the two different fluorescence signals from SMA-g-PA12&Pyrene and naphthylimide-C_12_ incorporated into PVC.

Herein, we report a new polymeric nucleating agent, SMA-g-PA12 (SP), formed by grafting PA12 onto SMA (Scheme S1), which integrates multiple advantages. On the one hand, PA12 crystallizes during processing, allowing PVC molecular chains to arrange themselves in an orderly manner on its surface, thereby reducing the energy required to form nuclei. On the other hand, the SMA-g-PA12&Pyrene (SPP) obtained after the modification with pyrene (Scheme S2) imparts distinct fluorescence to the crystalline regions of PA12. Additionally, we synthesize another fluorescent molecule, naphthylimide-C_12_ (Scheme S3), and incorporate it into PVC. The interfacial layer of the induced crystallization can be clearly observed through the two different fluorescent signals from PA12 and PVC, opening a new door for the applications of polymeric nucleating agents in PVC (Figure 1). Overall, this research highlights a polymeric nucleating agent that enables the induced crystallization of rigid PVC during processing, broadening the application scope of amorphous polymer. It also provides an effective means of visualizing the polymer interfacial layer, contributing to the development of polymer crystallization.

## 2. Materials and Methods

### 2.1. Materials

The synthesis and characterization of SMA-g-PA12 (SP), SMA-g-PA12&Pyrene (SPP), and naphthylimide-C_12_ were fulfilled according to the synthetic route of Schemes S1–S3 in the Supplementary Materials. All reagents and solvents were purchased with analytical purity, and used as purchased without further purification. The detailed information of all materials and instrument were provided in the Supplementary Materials.

### 2.2. Preparation of PVC Samples

#### 2.2.1. Preparation of PVC Films Containing Nucleating Agents with Different SMA Contents

Premixed method: A total of 1.5 phr PA12 or SP, 100 phr PVC and additives (4 phr zinc stearate, 4 phr calcium stearate, 3 phr acrylate copolymers (ACR-401), 0.5 phr stearic acid, 1.5 phr polyethylene wax (PE wax), and 5 phr dioctyl phthalate (DOP) were simultaneously premixed in a multi-functional pulverizer at 24,000 rpm/min for 60 s. Then, the above samples were premixed at 10,000 rpm/min for 4 min to obtain homogeneous premixed powders.

Processed method: Premixed powders were processed to 0.4 mm thick PVC films through an open mill at 180 °C for 10 m. The formula is given in Table 1.

#### 2.2.2. Preparation of PVC Strips with Different SP6% Contents

PVC and additives were mixed in same premixed method as above, and the amount of nucleating agent SP6% added is shown in Table 1. The PVC samples were placed in a mold and made into 4 mm thick strips through a plate vulcanizing machine.

#### 2.2.3. Preparation of PVC Films with Different SPP Contents

PVC and additives were mixed in the same premixed method as above, and the amount of nucleating agent SPP added is shown in Table 1. The premixed powders were passed through an open mill at 180 °C for 10 min to produce 25 mm thick PVC films. The formula is given in Table 1.

#### 2.2.4. Preparation of Fluorescent PVC Films with SPP and Naphthylimide-C_12_

PVC and additives were mixed in the same premixed method as above, in which 1.5 phr nucleating agent SPP and 0.02 phr naphthylimide-C_12_ were added. The premixed powders were passed through an open mill at 180 °C for 10 min to produce 25 mm thick fluorescent PVC films.

## 3. Results and Discussion

### 3.1. Induced Crystallization of PVC by SP

**Figure 2:**
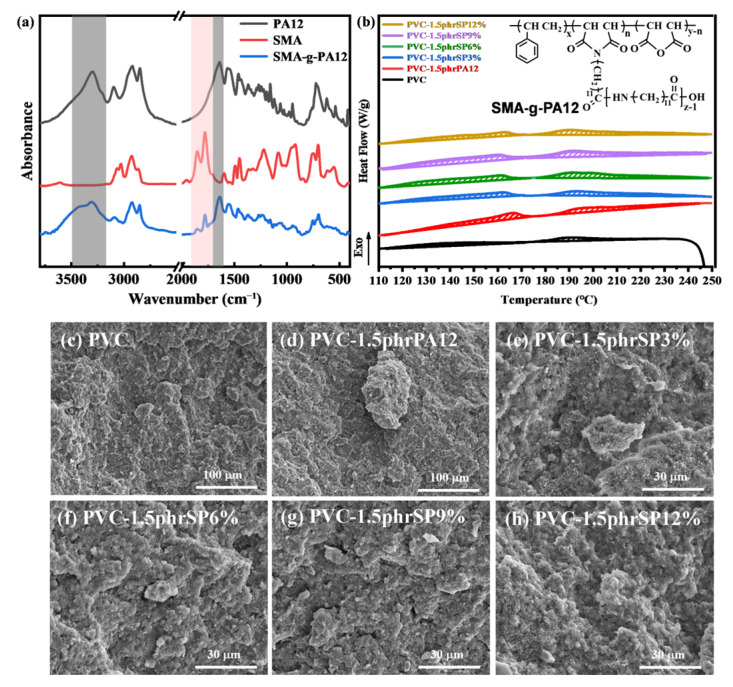
(**a**) FTIR images of PA12, SMA, and SMA-g-PA12. (**b**) Melting curves (inset figure is the chemical structure of SMA-g-PA12) and (**c**–**h**) SEM images of PVC containing 1.5 phr SP with different SMA contents.

Originally, the structure and properties of SMA-g-PA12 have been thoroughly characterized. Firstly, the structure of PA12, SMA, and SMA-g-PA12 were characterized using FTIR (Figure 2a). The characteristic peaks of SMA-g-PA12 appeared at 3315 cm^−1^ and 1637 cm^−1^, corresponding to the stretching vibration peaks of amide groups in PA12. And 1780 cm⁻¹ and 1856 cm⁻¹ were the stretching vibration peaks of the anhydride groups in SMA, indicating that PA12 had been successfully grafted onto SMA. Additionally, the compatibility, size, and crystallinity of SMA-g-PA12 had a very important influence on their crystallization-inducing effect. The size and crystallinity of SP with varying SMA content were investigated using scanning electron microscopy (SEM) and differential scanning calorimetry (DSC). The results indicated that SP6% exhibited an appropriate size and the highest crystallinity (Figures S4–S6 and Table S1).

Then, the induced crystallization of PVC containing 1.5 phr SP with different SMA contents was investigated. PVC exhibited two melting peaks associated with crystallization in melting curves (Figure 2a): a melting peak in the melting curves of secondary crystallization at around 110–175 °C and a melting peak of primary crystallization at around 175–248 °C [38]. Pure PVC degraded at 240 °C, while PVC containing SP remained stable at 250 °C, due to improvement in the induced crystallinity. The crystallinity of PVC was calculated using Equation S4 to exclude the influence of the crystallinity of SP and processing aids, as shown in Table 2. The crystallinity of PVC was significantly enhanced after the addition of the polymeric nucleating agent. The addition of 1.5 phr SP6% caused the crystallinity of PVC to peak at 11.6%, which represented an approximately 2.5-fold increase compared to PVC. Subsequently, the dispersion of SP in PVC was observed by SEM (Figure 2b). When 1.5 phr PA12 or 3% SP was added, poorly compatible nucleating agents measuring 30–100 μm were observed in PVC. As the content of SMA increased, the size of the SP decreased (Figure S4b) and their compatibility improved, resulting in no significant aggregates in PVC [39]. In SP12%, although the higher SMA content resulted in smaller particle sizes, it also led to reduced crystallinity, thereby weakening the induced crystallization effect on PVC. In summary, the content of the compatibilizer in SP had a significant impact on its size, crystallinity, and dispersion in PVC. SP6% demonstrated excellent crystallization-inducing effect on PVC due to its high crystallinity and good dispersion properties.

**Figure 3:**
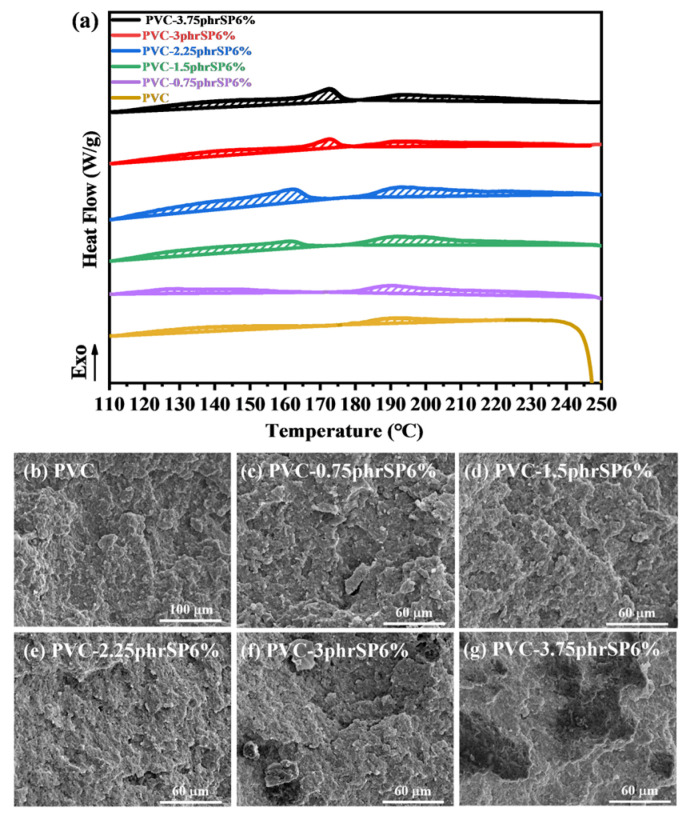
(**a**) Melting curves. (**b**–**g**) SEM images of PVC containing various SP6% contents.

Based on the excellent crystallization-inducing effect of SP6%, the PVC containing various SP6% contents was further investigated to achieve the optimal crystallization-inducing effect. The DSC results indicated that the crystallinity of PVC gradually increased with the increasing concentration of SP6% (Figure 3a, Table 3). The addition of 2.25 phr SP6% resulted in PVC exhibiting its highest crystallinity of 15.1%, which represented an approximately 3.3-fold increase compared to PVC (the XRD curves in Figure S8 are consistent with this result). The continued increase in SP6% content led to a significant decrease in the crystallinity of PVC. This was likely due to the extensive aggregation of the SP component, which was confirmed by the SEM results. As shown in Figure 3b, when the addition of SP6% to PVC was in the range of 0–2.25%, SP6% was well dispersed within PVC, and no aggregates were observed. In the PVC containing 3 phr and 3.75 phr SP6%, voids could be observed, corresponding to the poor compatibility causing SP6% aggregates to detach from the matrix. In contrast, at the larger addition amounts, SP6% exhibited good compatibility with PVC, avoiding aggregation, and effectively increasing the number of heterogeneous nucleation of PVC, thereby enhancing the overall crystallinity.

### 3.2. Mechanical Properties and Thermal Stability of PVC Containing SP

**Figure 4:**
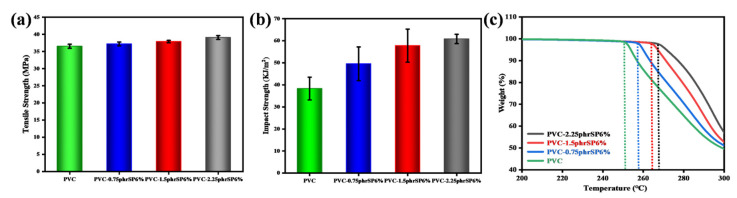
(**a**) Tensile strength; (**b**) impact strength; and (**c**) TG curves of PVC containing various SP6% contents.

After investigating the optimal induced crystallization effect of SP, the mechanical and thermal stability properties of PVC were studied in detail. As the SP contents increased, the tensile strength of PVC slightly improved from 36.70 MPa to 39.12 MPa (Figure 4a). At the same time, the presence of numerous highly electronegative chlorine atoms in the PVC molecular chains increased the polarity, which hindered the movement of the polymer chains [40]. As a result, rigid PVC exhibited relatively weak impact resistance. However, the impact strength of PVC with the addition of SP significantly increased as shown in Figure 4b. The impact strength of PVC-2.25phrSP6% was 61.03 kJ/m^2^, which represented an approximately 1.6-fold increase compared to PVC. Due to SP’s composition of highly tough PA12 and highly compatible SMA, along with the synergistic effect of the induced crystalline regions in PVC, a considerable toughening effect on rigid PVC was achieved [41].

Additionally, the thermal stability of PVC containing various SP6% contents also experienced a significant improvement. The TG curves showed that the apparent weight loss temperature of PVC-2.25phrSP6% was 267 °C, which was an increase of 17 °C compared to PVC. Therefore, as the crystallinity increased, the larger number of induced crystalline regions in PVC further restricted molecular chain movement, resulting in a higher degradation temperature for PVC [42].

### 3.3. Visual Observation of the Induced Crystallization of PVC

In the above study, the induced crystallization effect of SP enhanced the impact strength and degradation temperature of rigid PVC through a single and straightforward processing step. Among these factors, the compatibility of SP in PVC significantly impacted performance improvement [43], and characterizing the interfacial layer between SP and PVC facilitated a more direct investigation of their compatibility. However, the commonly used characterization method for observing polymer crystalline morphology, POM, could not effectively visualize the induced crystallization of PVC, as the processing of PVC required the addition of a large amount of crystalline processing aids. Thus, using two fluorescent sensors to localize SP and PVC, respectively, proved to be an effective means for observing the interfacial layer, which was also an innovative approach for observing polymer-induced crystallization morphology.

**Figure 5:**
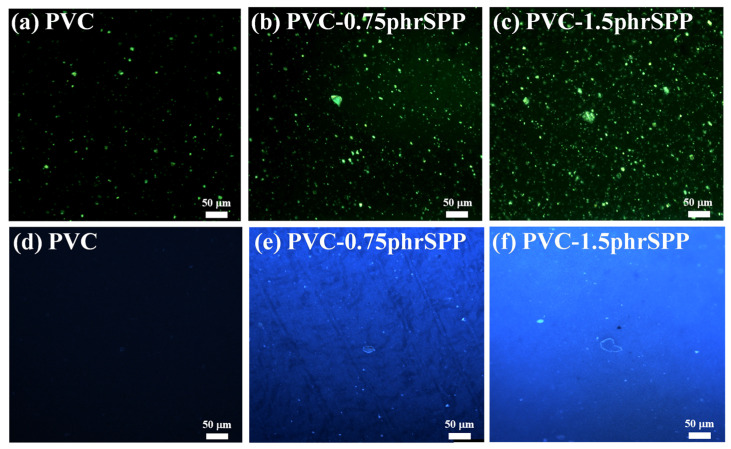
(**a**–**c**) POM images and (**d**–**f**) FM images of PVC containing various SPP contents.

After modifying SP6% with pyrene, SMA-g-PA12&Pyrene (SPP) not only exhibited excellent induced crystallization effect but also possessed fluorescent properties in its crystalline regions. The UV absorption and fluorescent emission of SPP were investigated (Figure S9) and the detailed photophysical properties are summarized in Table 4. Simultaneously, the crystalline regions of SPP were observed using FM and POM images (Figure S10). Furthermore, the PVC containing various SPP contents were also observed in detail via POM and FM, as shown in Figure 5. In the POM images, the crystallization of processing aids was observed, which appeared non-fluorescent in the FL images (Figure 5a,d). But both the induced crystallization and fluorescent emission of PVC significantly increased with higher SPP content. Therefore, fluorescent sensors could eliminate the interference of PVC processing aids on the observation of induced crystallization morphology.

**Figure 6:**
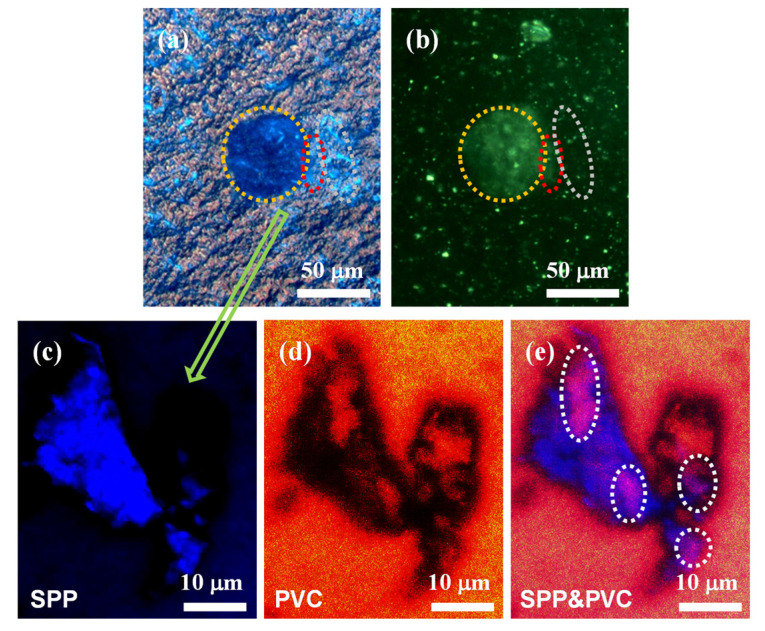
(**a**) FM image; (**b**) POM image; and (**c**–**e**) CLSM images of PVC containing SPP and naphthylimide-C_12_. (**c**) *λ*_abs-CLSM_ = 405 nm, *λ*_em-CLSM_ = 370–470 nm; (**d**) *λ*_abs-CLSM_ = 488 nm, *λ*_em-CLSM_ = 500–600 nm; (**e**) *λ*_abs-CLSM_ = 405 nm and 488 nm, *λ*_em-CLSM_ = 370–470 nm and 500–600 nm.

The above FM images of PVC containing SPP only achieved fluorescent localization of the crystalline regions of SPP, but observing the interfacial layer proved to be more challenging. We designed and synthesized another fluorescent sensor, naphthylimide-C_12_, which exhibited distinct photophysical properties compared to pyrene, to impart fluorescent characteristics to the PVC matrix. The UV absorption and fluorescent emission of naphthylimide-C_12_ were investigated (Figure S11), which is summarized in Table 4 for better comparative analysis. The FM image of PVC containing SPP and naphthylimide-C_12_ showed that the light blue fluorescent PVC aggregated next to the dark blue fluorescent SPP and the interfacial layer between the two exhibited a mixed fluorescence of both colors (Figure 6a). And the crystalline regions of SPP and the interfacial layer observed by POM completely matched the FM results. To more clearly observe this interfacial layer of PVC-induced crystallization, CLSM with a higher magnification was employed. Based on the photophysical properties of SPP and naphthalimide-C_12_ (Table 4), we observed the fluorescence of SPP and PVC at different excitation and emission wavelengths, with their fluorescent regions designated in blue and red, respectively (Figure 6c,d). When their fluorescence was observed simultaneously, multiple regions of overlapping blue and red were clearly visible, indicating the interfacial layer of induced crystallization formed by the mutual diffusion of the SPP and PVC polymer chains. The strategy of using two different fluorescent sensors for the visualization of the interfacial layer in both the polymeric nucleating agent and the polymer matrix provided valuable support for the design of novel nucleating agents and their subsequent applications.

## 4. Conclusions

In summary, we propose an induced crystallization strategy for significantly enhancing the physical properties of polyvinyl chloride through a customized polymer nucleating agent, formed by grafting polyamide12 onto styrene–maleic anhydride copolymer, which offers a low melting point, excellent compatibility, and high crystallinity. The importance of this strategy lies in its ability to effectively improve the crystallinity of polyvinyl chloride through a single and straightforward processing step, which directly correlates with enhanced impact strength and elevated degradation temperature. Additionally, the polymeric nucleating agent and polyvinyl chloride can incorporate two fluorescent sensors directly, enabling in situ visualization of the interfacial layer. This represents a key breakthrough in the characterization of polymer crystallization. We expect that the visualization of the polymer interfacial layer will inspire further research and the development of efficient polymeric nucleating agents, facilitating significant advancements in the applications of amorphous polymers in various industries.

## Figures and Tables

**Table 1:** Formula of PVC samples with different polymeric nucleating agents.

Samples	PA12	SP3%	SP6%	SP9%	SP12%	SPP
PVC	/	/	/	/	/	/
PVC-1.5phrPA12	1.50 phr	/	/	/	/	/
PVC-1.5phrSP3%	/	1.50 phr	/	/	/	/
PVC-1.5phrSP6%	/	/	1.50 phr	/	/	/
PVC-1.5phrSP9%	/	/	/	1.50 phr		/
PVC-1.5phrSP12%	/	/	/	/	1.50 phr	/
PVC-0.75phrSP6%	/	/	0.75 phr	/	/	/
PVC-1.5phrSP6%	/	/	1.50 phr	/	/	/
PVC-2.25phrSP6%	/	/	2.25 phr	/	/	/
PVC-3phrSP6%	/	/	3.00 phr	/	/	/
PVC-3.75phrSP6%	/	/	3.75 phr	/	/	/
PVC-0.75phrSPP	/	/	/	/	/	0.75 phr
PVC-1.5phrSPP	/	/	/	/	/	1.50 phr

where SP is SMA-g-PA12. SP3%, SP6%, SP9%, and SP12% refer to the content of SMA in SMA-g-PA12 being 3 wt%, 6 wt%, 9 wt%, and 12 wt%. SPP is SMA-g-PA12&Pyrene. PVC-1.5phrSP3% refers to a PVC sample that contains 1.5 phr of SP3%, and other samples are named in the same way.

**Table 2:** DSC data of PVC containing 1.5 phr SP with different SMA contents.

Sample	*T*_m2_/°C	*T*_m1_/°C	Δ*H*_m2_/J·g^−1^	Δ*H*_m1_/J·g^−1^	$x_{c}$
PVC	146.2	188.5	1.5	0.9	4.6%
PVC-1.5phrPA12	167.0	193.0	2.7	2.5	9.9%
PVC-1.5phrSP3%	163.1	191.8	2.8	2.8	10.7%
PVC-1.5phrSP6%	161.5	191.5	3.1	3.2	11.6%
PVC-1.5phrSP9%	161.0	190.9	2.5	2.8	10.1%
PVC-1.5phrSP12%	163.0	189.8	2.4	2.3	9.0%

where *T*_m1_ is the melting peak of primary crystallization, *T*_m2_ is the melting peak of secondary crystallization, Δ*H*_m1_ is the enthalpy of melting of primary crystallization, and Δ*H*_m2_ is the enthalpy of the melting for secondary crystallization.

**Table 3:** DSC data of PVC containing various SP6% contents.

Sample	*T*_m2_/°C	*T*_m1_/°C	Δ*H*_m2_/J·g^−1^	Δ*H*_m1_/J·g^−1^	c_c_
PVC	146.2	188.5	1.5	0.9	4.6%
PVC-0.75phrSP6%	146.7	189.9	1.6	3.0	8.8%
PVC-1.5phrSP6%	161.5	191.5	3.1	3.1	11.7%
PVC-2.25phrSP6%	162.5	192.7	4.4	3.6	15.1%
PVC-3phrSP6%	164.2	192.0	2.6	1.8	8.2%
PVC-3.75phrSP6%	167.1	193.9	3.5	2.1	10.3%

**Table 4:** Summary of photophysical properties of SPP and naphthalimide-C_12_.

Sample	*λ*_abs_/nm	*λ*_em_/nm	*λ*_abs-CLSM_/nm	*λ*_em-CLSM_/nm
SPP	300–480	420–490	405	370–470
Naphthalimide-C_12_	380–500	500–600	488	500–600

## Data Availability

The data that support the findings of this study are available from the corresponding author upon reasonable request.

## Supplementary Materials

The following supporting information can be downloaded at: https://www.mdpi.com/article/10.3390/polym16223147/s1, Figure S1: 1H NMR spectra of naphthalimide-C12; Figure S2: 13C NMR spectra of naphthalimide-C12; Figure S3: LC-MS spectra of naphthalimide-C12; Figure S4: (a) FTIR images of SP3%, SP6%, SP9% and SP12%. (b–f) SEM images of PA12, SP3%, SP6%, SP9%, SP12%.; Figure S5: XRD curves of PA12 and SP3%, SP6%, SP9%, SP12%; Figure S6: DSC curves of PA12 and SP3%, SP6%, SP9%, SP12%; Figure S7: FTIR images of SP6%, 1-aminopyrene and SPP; Figure S8: XRD curves of PVC, PVC-PA12 and PVC containing 1.5 phr SP with different SMA contents; Figure S9: (a) UV absorption of SPP and 1-aminopyrene, fluorescent emission curves of (b) 1-aminopyrene and (c) SPP, λex = 405 nm.; Figure S10: demonstrated that naphthylimide-C12 exhibited UV absorption at both 405 nm and 488 nm, while its fluorescent emission remained consistent across both excitation wavelengths.; Figure S11: (a) UV absorption of naphthylimide-C12, fluorescence emission curves of naphthylimide-C12, (b) ex = 405 nm and (c) ex = 488 nm (inset figure is FM image of naphthylimide-C12); Scheme S1 Synthetic Route of SMA-g-PA12; Scheme S2 Synthetic Route of SMA-g-PA12&Pyrene; Scheme S3 Synthetic route of naphthalimide-C_12;_ Table S1: Characteristic parameter values of PA12 and SP [44,45,46,47,48,49].
