# Supplementary material for: In Situ Fluorescent Visualization of the Interfacial Layer of Induced Crystallization in Polyvinyl Chloride
Source: Polymers (Basel). 2024 Nov 12;16(22):3147. doi: 10.3390/polym16223147 (PMC11597979; doi:10.3390/polym16223147)
Supplement: Supplementary file 1 [file polymers-16-03147-s001.zip › polymers-3280517-supplementary.pdf]

Supplementary information

In situ Fluorescent Visualization of the Interfacial Layer of Induced Crystallization in Polyvinyl Chloride

Zhihang An,[a] Renping Liu,[b] Zhenhao Dai,[a] Jiaping Liu,[a] Jiaying Du,[a] Zhongyi Sheng*[a] and Heyang Liu*[ac]

[a] College of Biological & Chemical Engineering, Zhejiang University of Science and Technology, Hangzhou, 310023, P. R. China

[b] Zhejiang Wazam New Materials Co., Ltd., Hangzhou, 311121, P. R. China

[c] College of Environmental and Natural Resources, Zhejiang University of Science and Technology, Hangzhou, 310023, P. R. China

* Corresponding authors. Email: shengzyhz@zust.edu.cn; heyang.liu@zust.edu.cn

Table of Contents

1. Synthesis and Characterization..1

2. Results and Discussion 10

1. Synthesis and Characterization

1.1 Materials

Polyvinyl chloride (PVC) was supplied by Tianye Group Co. Lt. (Xinjiang, China). Acrylate copolymers (ACR-401) was supplied by Rike Group Co. Lt. (Shandong, China). Zinc stearate, calcium stearate, stearic acid, polyethylene wax (PE

wax) and dioctyl phthalate (DOP) were supplied by Macklin Reagent (Shanghai, China). Polyamide 12 (PA12) was supplied by Evocnik Group Co. Lt. (Shanghai, China). Styrene-maleic anhydride copolymer (SMA, molar ratio of ST/MAH=3:1, melting point 165℃) was supplied by Hua Wen Evocnik Group Co. Lt. (Jiaxing, China). Phenol (AR), ethanol (99.7%), acetone (AR), 1-aminepyrene (AR), 4-bromine-1,8-naphthalene anhydride (AR), isoamylol (AR), 3-(dimethylamino) propiononitrile (AR) and 1,12-diaminododecane (AR) were supplied by Macklin Reagent (Shanghai, China).

1.2 Instrumentation

The instruments for processing PVC include open mill SKL120 and vulcanizing press SPY300. The melting curves of the PVC were measured by Netzsch differential scanning calorimeter (DSC). The tensile strength and impact strength of PVC were measured using New Sansi's Universal Material Tester and Pendulum Impact Tester respectively. The decomposition temperature of PVC was measured by a Mettler thermogravimetric analyser (TGA). Scanning electron microscope (SEM) images were recorded on a Hitachi S-3700N. Fluorescence microscope (FM), polarizing microscope (POM) and confocal laser scanning microscope (CLSM) photos were obtained using Olympus BX53F, Yunke 59XB and Zeiss LSM 900 with Airyscan2. UV-vis and fluorescence spectra were obtained using a Shimadzu UV2600 UV-vis-NIR spectrophotometer and a Hitachi F-4500 fluorescence spectrometer. Multi-function shredder and micropulverizer were belong to Baixin LG-01 and Tuoyan FW80. The structure of the nucleating agent was measured by Fourier

Transform Infrared Spectrometer (FTIR). ^{1}H and ^{13}C nuclear magnetic resonance (NMR) spectra were recorded on Bruker AVANCE III (500MHz) NMR spectrometer in $CDCl_3$ using tetramethylsilane (TMS) as an internal standard. Mass spectra were recorded on a Shimadzu Liquid Chromatography-Time of Flight Mass Spectrometry. UV-vis and fluorescence spectra were obtained using a Shimadzu UV2600 UV-vis-NIR spectrophotometer and a Hitachi F-4500 fluorescence spectrometer.

1.3 Synthesis of SMA-g-PA12

In a three-necked flask fitted with a condenser tube, PA12, phenol and ethanol were added. The sample was magnetically stirred at 80 °C for 30 min, resulting in a white suspension. Then SMA was added and refluxed at 80 °C for 10 h. After refluxing, the suspension was subjected to reduced pressure distillation at 120 °C to remove the phenol. The resulting solid was crushed using a mortar and pestle to produce white powder, designated SMA-g-PA12 (SP). To purify the powder, residual phenol was removed by washing with ethanol, and any unreacted SMA was eliminated by washing with acetone. The final SP powder was then dried and sieved to obtain a nucleating agent with a particle size of less than 54 μm. The content of SMA in SMA-g-PA12 was calculated using Equation S1.

$$\text{content of SMA} = \frac{m_{SP} - m_{PA12}}{m_{PA12}} \qquad \text{Equation S1}$$

The m in equation S1 is the mass.

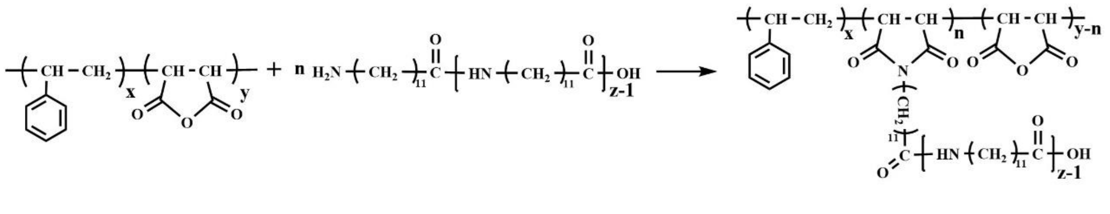

Scheme S1 Synthetic Route of SMA-g-PA12

1.4 Synthesis of SMA-g-PA12&Pyrene (SPP)

In a three-necked flask fitted with a condenser tube, 11.62 g of SMA-g-PA12 (containing 6 wt% SMA) and 360.0 g phenol were added. The mixture was magnetically stirred at 80 ℃ for 30 minutes to form a suspension. The 0.83 g 1-aminopyrene was dissolved in 50 mL ethanol to form a solution, which was added to the suspension and refluxed at 80 ℃ for 10 hours. Then the suspension was distilled under reduced pressure at 130 ℃ to remove the phenol, and the resulting solids are pulverized and washed with a large amount of ethanol. The SPP obtained after drying contains 4.22 wt% of 1-aminopyrene. Solids were sieved to obtain fluorescent nucleators SMA-g-PA12&Pyrene (SPP) with particle sizes below 54 μm. SP and SPP were separately moulded into 0.1 mm thick transparent films using a plate vulcanisation machine.

$$\text{content of 1-aminopyrene} = \frac{m_{SPP} - m_{SP}}{m_{SP}} \qquad \text{Equation S2}$$

The m in equation S2 is the mass, wt%.

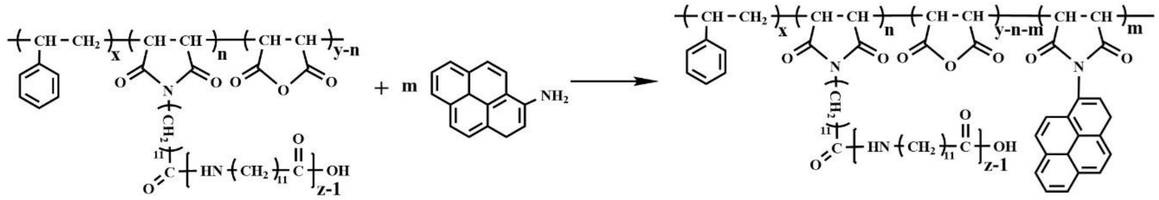

Scheme S2 Synthetic Route of SMA-g-PA12&Pyrene

1.5 Synthesis of Naphthylimide-C_{12}

Synthesis of 4-dimethylamino-1,8-naphthalenimide:[1] 4-bromo-1,8-naphthimide

(11 g, 39.7 mmol), 3-(dimethylamino) propionitrile (16 g, 163 mmol) and 280 mL isopentyl alcohol were refluxed at 132 °C for 12 h. The mixture was filtered to obtain orange solids, which were rinsed with deionised water and isohexane to get 4-dimethylamino-1,8-naphthimide (5.0 g, 50 %).

Synthesis of naphthylimide-C_{12}: 4-Dimethylamino-1,8-naphthimide (1.21 g, 1 mmol), 1,12-diaminododecane (1.2 g, 1.2 mmol) and ethanol 30 mL were refluxed at 80 °C for 12 h, and cooled to precipitate yellow solids. The naphthylimide-C_{12} (0.53 g，25 %) was obtained by column chromatography purification with the eluent of EA/PE = 1/5. ^{1}H NMR (400 MHz, Chloroform-*d*) δ 8.59 (dd, *J* = 7.2, 1.2 Hz, 1H), 8.50 (d, *J* = 8.1 Hz, 1H), 8.45 (dd, *J* = 8.5, 1.2 Hz, 1H), 7.68 (dd, *J* = 8.5, 7.3 Hz, 1H), 7.14 (d, *J* = 8.2 Hz, 1H), 4.21 - 4.14 (m, 2H), 3.12 (s, 6H), 1.74 (q, *J* = 7.4 Hz, 2H), 1.42-1.31 (m, 4H), 1.25 (s, 4H). ^{13}C NMR (100MHz, Chloroform-*d*) δ 164.5, 164.0, 132.5, 131.0, 130.9, 125.3, 125.0, 123.2, 113.5, 77.2, 44.8, 40.3, 29.6, 29.5, 29.4, 28.2, 27.2. LC-MS: theoretical value is $[M+H]^+$=647.37，The measured value is 647.37，The structure of the resulting product corresponds to the designed structure.

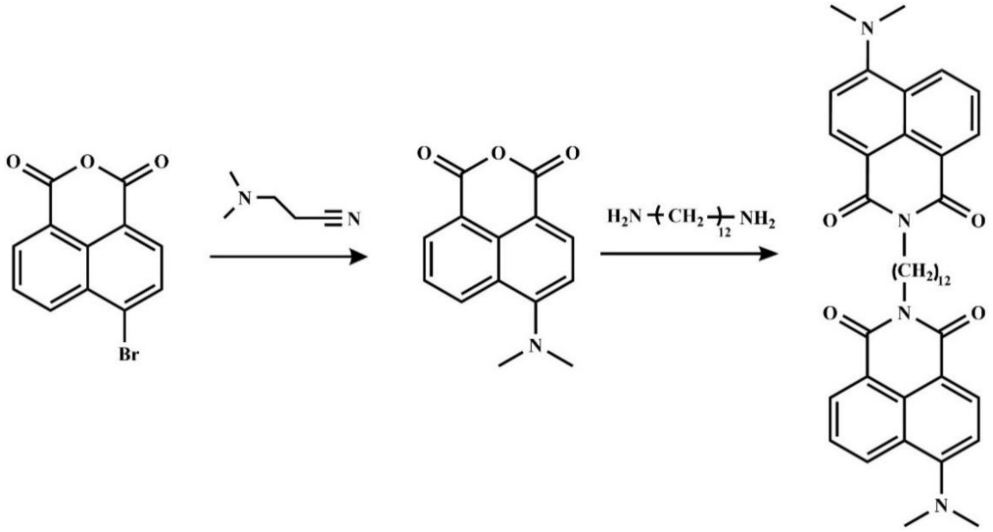

Scheme S3 Synthetic route of naphthalimide-C_{12}

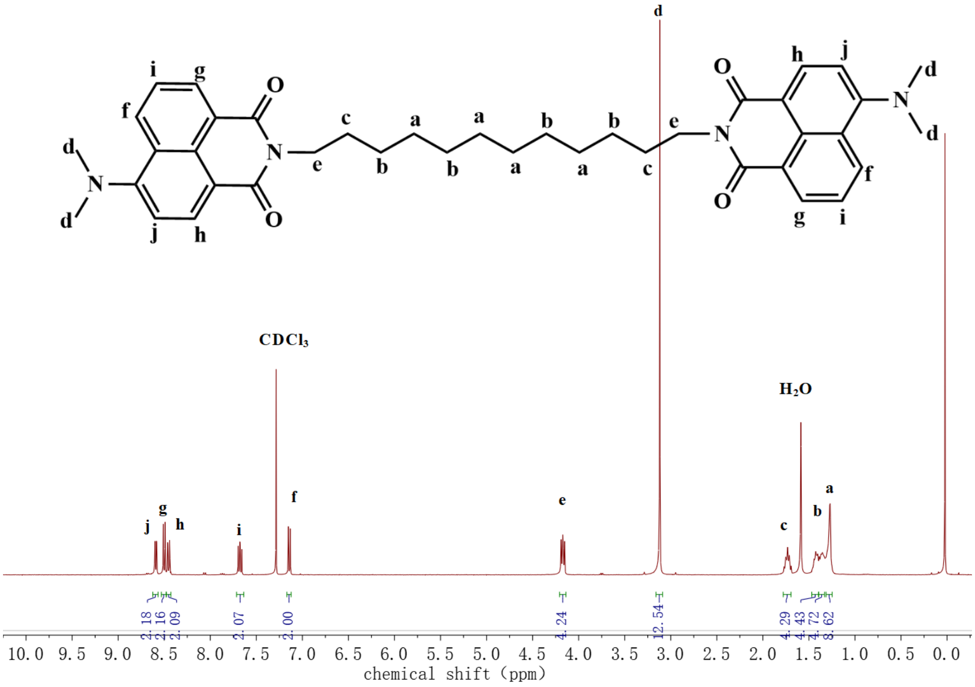

Figure S1 ^{1}H NMR spectra of naphthalimide-C12

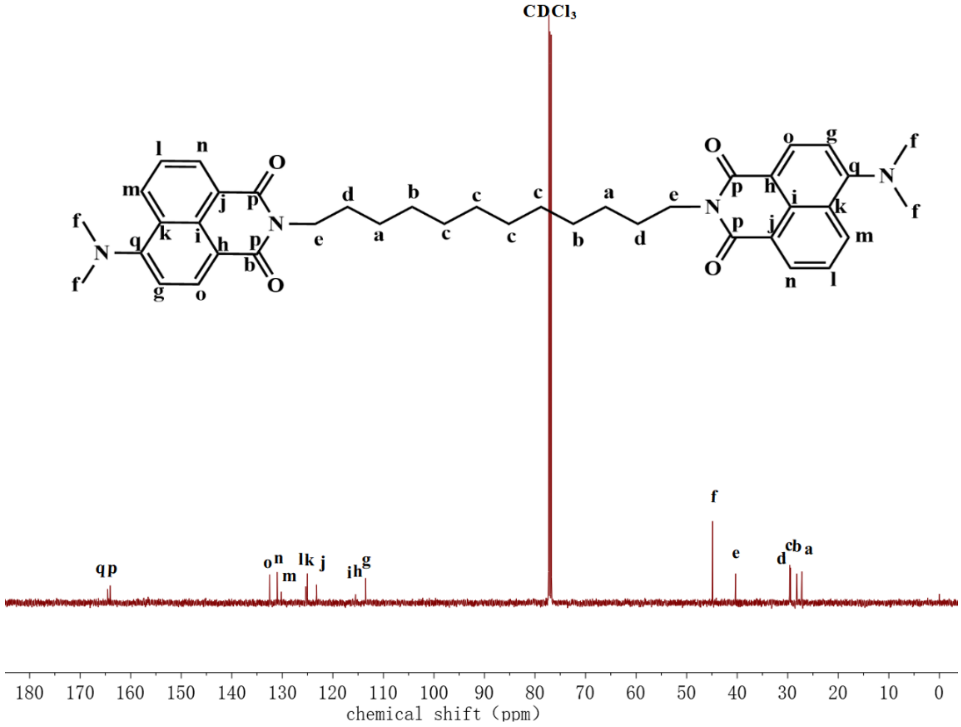

Figure S2 ^{13}C NMR spectra of naphthalimide-C12

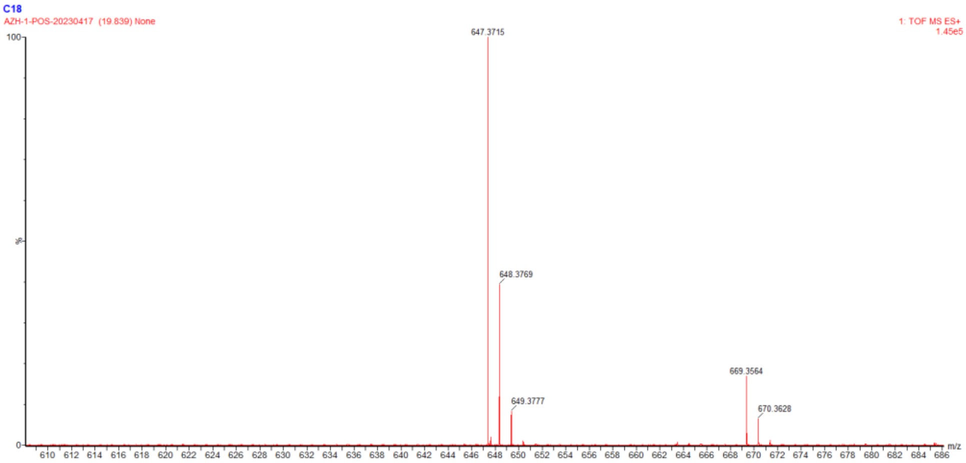

Figure S3 LC-MS spectra of naphthalimide-C_{12}

1.6 Characterization Method

1.6.1 Fourier Transform Infrared Spectrometer (FTIR)

To characterize the chemical structure of SPP, powder samples of SP and SPP were analyzed using KBr pellet compression. The spectral data were collected over a scan range of 4000 to 400 cm^{-1} with a resolution of 4 cm^{-1}.

1.6.2 Differential Scanning Calorimetry (DSC)

The polymeric nucleating agents were analyzed using a DSC apparatus under a nitrogen atmosphere to determine the melting and crystallization behavior. The temperature was first increased from room temperature to 200 °C at a ramp rate of 30 °C/min and held at 200 °C for 3 minutes. The temperature was then decreased back to room temperature at the same ramp rate and held for 3 minutes before being reheated to 200 °C at a ramp rate of 30 °C/min. The second heating curve was used for the analysis of the melting behavior. The crystallinity was calculated by the formula:

$$x_c = \frac{\Delta H_m}{\Delta H_\mu} \qquad \text{Equation S3}$$

ΔH_m is the melting enthalpy, $J \cdot g^{-1}$. ΔH_μ is the theoretical crystallization enthalpy of PA12, 209.0 $J \cdot g^{-1}$,[2] and x_c is the crystallinity of PA12 or SP, %.

Melting curves of PVC were analyzed using a DSC apparatus under nitrogen atmosphere. Temperature rise rate 20 °C/min from 25 °C to 250 °C. The crystallinity was calculated by the formula:

$$x_c = \frac{\Delta H_m}{\Delta H_0} \omega_{PVC} \qquad \text{Equation S4}$$

where ΔH_m was the melting enthalpy of the PVC sample, $\Delta H_m = \Delta H_{m1} + \Delta H_{m2}$, $J \cdot g^{-1}$. ΔH_0 was the theoretical crystallisation enthalpy of PVC, 43.9 $J \cdot g^{-1}$.[3] The x_c was the PVC crystallinity of the sample, %. The ω_{PVC} was the mass fraction of PVC.

1.6.3 Scanning Electron Microscope (SEM)

The PVC samples were brittle fractured using liquid nitrogen and then mounted on the conductive adhesive of the test rig for gold coating. The cross-sectional morphology of the PVC samples was subsequently examined using a scanning electron microscope.

1.6.4 UV-vis-NIR Spectrophotometer

The 1-aminopyrene and naphthylimide-C_{12} powders were applied to the light-transmitting side of a quartz cells and an empty quartz cell was used as a standard sample with a scanning range of 300-600 nm.

Transparent films of SP, SPP and PVC were placed in a quartz cells and an empty quartz cell with a scanning range of 300-600 nm.

1.6.5 Fluorescence Spectrometer

The 1-Aminepyrene and SPP were measured with an excitation wavelength of 405 nm and a fluorescent emission wavelength range of 300-800 nm.

Naphthalimide-C_{12} was measured with an excitation wavelength of 488 nm and a fluorescent emission wavelength range of 300-800 nm.

PVC, PVC-0.75phrSPP and PVC-1.5phrSPP were measured with an excitation wavelength of 328 nm and a fluorescent emission wavelength range of 300-800 nm.

1.6.6 Fluorescence Microscope

SPP powder and various PVC films were placed on the stage. The samples were observed under UV excitation with an excitation wavelength range of 340-390 nm, using a 10× eyepiece magnification.

1.6.7 Polarizing Microscope

SPP powder and various PVC films were placed on a carrier table and observed under a 10× eyepiece magnification to examine the crystalline regions.

1.6.8 Confocal Laser Scanning Microscope

The PVC films were placed on a carrier stage and observed using a 40× objective lens to examine the crystalline regions.

1.6.9 Mechanical Performance

Impact specimens without notches were prepared according to the national standard GB/T 1843-2008. Impact testing was then conducted using a pendulum impact tester. Additionally, a 4 mm thick dumbbell-shaped specimen strip was prepared in accordance with GB/T 1040.2-2022. Tensile testing was performed using a universal materials testing machine at a tensile speed of 20 mm/min.

1.7.0 Thermal Stability

A thermogravimetric analyzer was used to assess the thermal stability of the PVC samples. The samples were heated from 50 °C to 800 °C with a nitrogen flow rate of 20 mL/min.

2. Results and Discussion

SEM results indicated that the size of the nucleating agent decreased as the SMA content increased. This reduction is attributed to the improved dispersion of PA12 with grafted SMA in the mixed solvent of phenol and ethanol. Additionally, a higher SMA content led to increased grafting of PA12 onto the anhydride groups, resulting in more effective dispersion of PA12.[4]

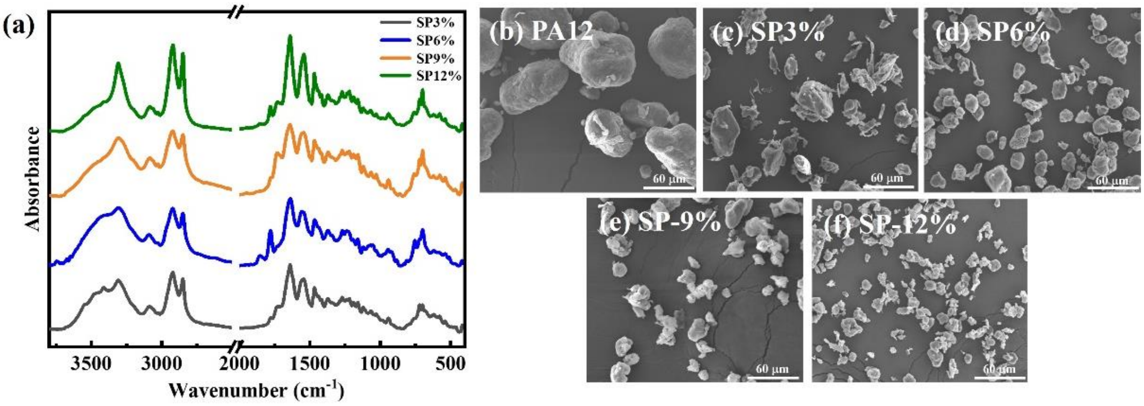

Figure S4 (a) FTIR images of SP3%, SP6%, SP9% and SP12%. (b)-(f) SEM images of PA12, SP3%, SP6%, SP9%, SP12%.

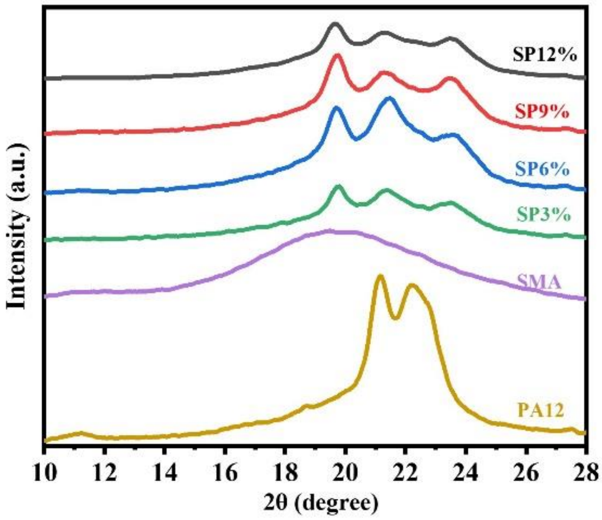

Figure S5 XRD curves of PA12 and SP3%, SP6%, SP9%, SP12%

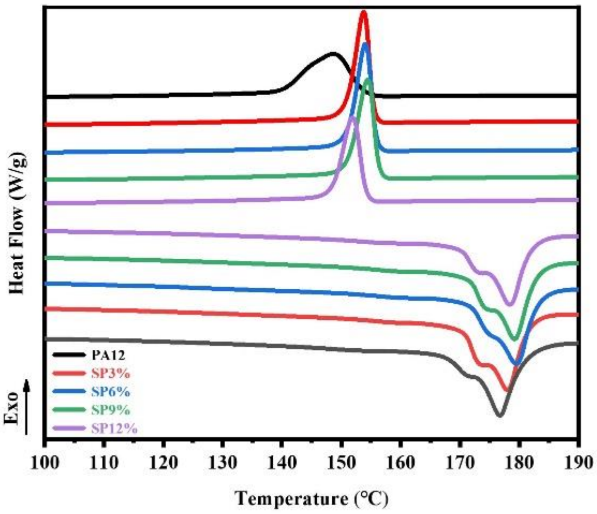

Figure S6 DSC curves of PA12 and SP3%, SP6%, SP9%, SP12%

The melting and crystallization profiles of SP with varying SMA contents were analyzed using DSC. The impact of SMA content on the crystalline properties of SP was illustrated in Figure S6 and Table S1. After grafting SMA onto PA12, both the melting peak temperature and the crystallization peak temperature of SP increased. Additionally, the overall crystallization rate of SP improved, the degree of subcooling

decreased, and crystallization became easier.[5] Notably, the crystallinity of the nucleating agent first increased and then decreased with higher SMA content. This trend is attributed to the influence of SMA on the regularity of the PA12 molecular chain arrangement.[6] When PA12 was grafted with larger amounts of SMA, the PA12 molecular chains became more dispersed, leading to decreased molecular chain alignment. Consequently, at an SMA content of 6%, the crystallinity of the nucleating agent reached a maximum value of 28.4%.

Table S1 Characteristic parameter values of PA12 and SP

Sample	Heating	Cooling		$(T_0$-$T_c)$/°C	$(T_m$-$T_c)$°C	ΔH_m/J·g^{-1}	x_c
	T_m/°C	T_c/°C	T_0/°C				
PA12	176.7	148.6	156.5	7.9	28.1	57.8	27.6%
SP3%	178.0	153.8	157.2	3.4	24.3	58.9	28.2%
SP6%	179.5	154.0	157.5	3.5	25.5	59.2	28.4%
SP9%	179.2	154.4	158.1	3.7	24.8	59.0	28.2%
SP12%	178.3	151.9	156.0	4.1	26.4	52.4	25.0%

Where T_m was the melting point of the more perfect crystal type. T_0 was the beginning crystallisation temperature. T_c was the crystallisation temperature, ΔH_m was the enthalpy of melting. T_0-T_c represented the overall rate of the crystallization process; a smaller value indicates a higher overall crystallization rate. And T_m-T_c represented the degree of supercooling; a smaller value indicates easier crystallization.

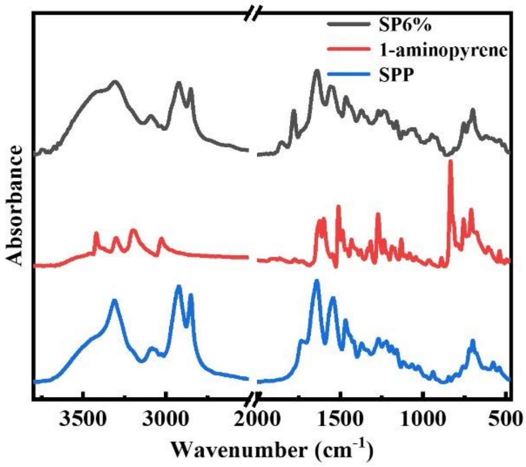

Figure S7 FTIR images of SP6%, 1-aminopyrene and SPP

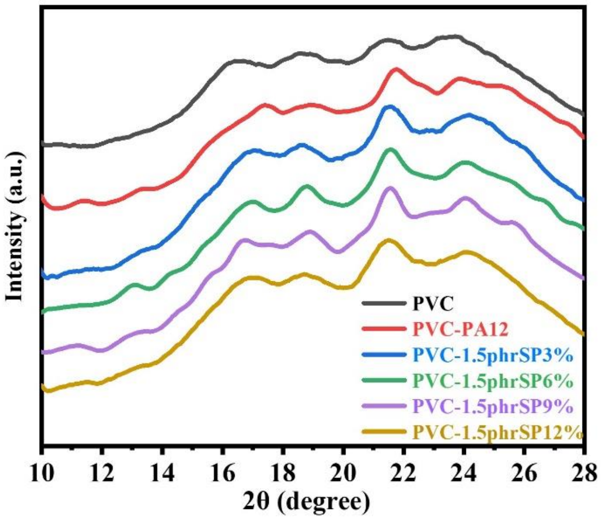

Figure S8 XRD curves of PVC, PVC-PA12 and PVC containing 1.5 phr SP with different SMA contents

The FTIR curve of SPP showed that the carbonyl peaks at 1779 cm^{-1} and 1852 cm^{-1} disappeared which were present in SP6%. The characteristic peak of C=O appeared at 1730 cm^{-1}. This indicates that the anhydride groups in SMA were nearly depleted after the grafting of 1-aminopyrene. Consequently, the structure of SPP was

altered, confirming that SP6% was successfully grafted with 1-aminopyrene.

To prepare for the FM and CLSM tests, the SPP was analyzed for UV absorption and fluorescence emission wavelengths. Figure S9a showed that the UV absorption wavelength range of SPP extended from 300 to 480 nm. The UV absorption spectra of 1-aminopyrene exhibited peaks near 360 nm, 380 nm, and 400 nm, corresponding to the π-π* transitions. The maximum absorption wavelength of SPP shifted from 360 nm to 343 nm, indicating a blue shift. This shift occurred because SPP was less planar than 1-aminopyrene, resulting in a reduced π-electron conjugation system in the pyrene unit. The electrons became delocalized over multiple atoms, increasing the π-π* transition energy and resulting in a blue shift of the maximum absorption wavelength in the UV absorption spectrum.[7]

Since the excitation wavelengths for the CLSM were fixed at 405 nm, 488 nm, and 561 nm, the excitation wavelength used for SPP was 405 nm. The fluorescent emission wavelength range of SPP was 420-490 nm (Figure S9c). Additionally, the fluorescent emission wavelength of SPP was blue-shifted, because the decrease in the degree of conformational planarity of 1-aminopyrene caused by the binding of the polymer chain. This reduction in planarity diminished the π-electronic conjugation system of the pyrene unit, leading to an increase in the energy of the excited state electrons and, consequently, a higher π*-π transition energy.

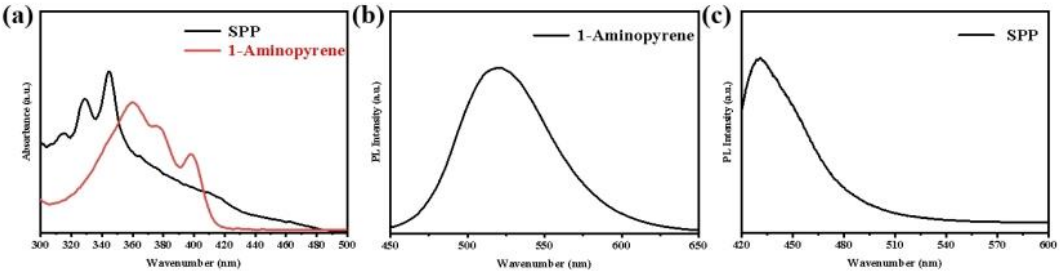

Figure S9 (a) UV absorption of SPP and 1-aminopyrene, fluorescent emission curves of (b) 1-aminopyrene and (c) SPP, λ_{ex}=405 nm.

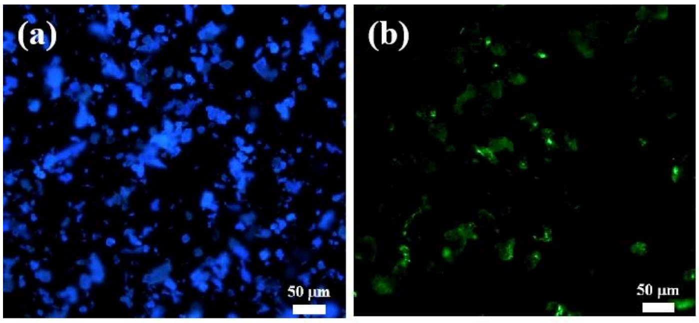

Figure S10 (a) FM and (b) POM images of SPP

Figure S11 demonstrated that naphthylimide-C12 exhibited UV absorption at both 405 nm and 488 nm, while its fluorescent emission remained consistent across both excitation wavelengths.

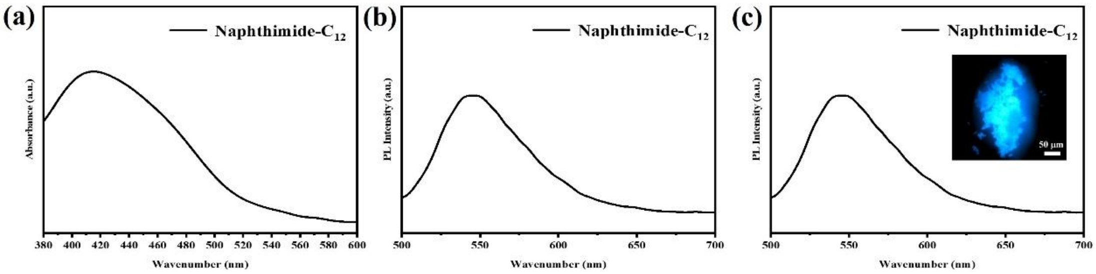

Figure S11 (a) UV absorption of naphthylimide-C_{12}, fluorescence emission curves of naphthylimide-C_{12}, (b) λ_{ex}=405 nm and (c) λ_{ex}=488 nm (inset figure is FM image of naphthylimide-C_{12}).

References

1. Kollár, J.; Hrdlovič, P.; Chmela, Š.; Sarakha, M.; Guyot, G. Synthesis and transient absorption spectra of derivatives of 1,8-naphthalic anhydrides and naphthalimides containing 2,2,6,6-tetramethylpiperidine; triplet route of deactivation. *J. Photoch. Photobio. A.* **2005**, *170*, 151-159. 10.1016/j.jphotochem.2004.07.021
2. Ma, N.; Liu, W.; Ma, L.; He, S.; Liu, H.; Zhang, Z.; Sun, A.; Huang, M.; Zhu, C. Crystal transition and thermal behavior of nylon 12. *e-Polymers* **2020**, *20*, 346-352. 10.1515/epoly-2020-0039
3. Zou, J.; Su, L.; You, F.; Chen, G.; Guo, S. Dynamic rheological behavior and microcrystalline structure of dioctyl phthalate plasticized poly(vinyl chloride). *J. Appl. Polym. Sci.* **2011**, *121*, 1725-1733. 10.1002/app.33765
4. Benhamou, M.; Himmi, M.; Kaidi, H. Induced force between colloidal particles with end-grafted polydisperse polymer chains: The role of the grafting mode. *J. Mol. Liq.* **2017**, *230*, 337-343. 10.1016/j.molliq.2016.12.114
5. Song, X.; Zhou, R.; Wu, Z.; Kang, J. Exploring the effects of stereo-defect distribution on the crystallization kinetics of isotactic polypropylene/cellulose nanocrystals composites. *Soft Mater.* **2019**, *17*, 375-382. 10.1080/1539445X.2019.1610893
6. Wang, C.; Sumida, A.; Adachi, Y.; Imoto, H.; Naka, K.; Ohshita, J. Group 15 element (As, Sb, Bi)-substituted bibenzofurans with noncovalent conformational locks for enhanced planarity. *Organometallics* **2023**, *42*, 3397-3404. 10.1021/acs.organomet.3c00373
7. Qi, L.; Zhu, Q.; Cao, D.; Liu, T.; Zhu, K.R.; Chang, K.; Gao, Q. Preparation and properties of stereocomplex of poly(lactic acid) and its amphiphilic copolymers containing glucose groups. *Polymers* **2020**, *12*, 760. 10.3390/polym12040760
